# Accuracy of Cup Placement Angle, Leg Lengthening, and Offset Measurement Using an AR-Based Portable Navigation System: Validation in Supine and Lateral Decubitus Positions for Total Hip Arthroplasty

**DOI:** 10.3390/medicina60101721

**Published:** 2024-10-21

**Authors:** Yusuke Ozaki, Takeaki Yamamoto, Satomi Kimura, Toru Kasai, Rintaro Niki, Hisateru Niki

**Affiliations:** 1 Department of Orthopaedic Surgery, St. Marianna University School of Medicine, 2-16-1 Sugao, Miyamae, Kawasaki 216-8511, Japan; yusuke.ozaki@marianna-u.ac.jp (Y.O.); satomi.kimura@marianna-u.ac.jp (S.K.); rintaro.niki@marianna-u.ac.jp (R.N.); h2niki@marianna-u.ac.jp (H.N.); 2 Department of Orthopedic Surgery, Yokohama Shinmidori Hospital, Yokohama 226-0025, Japan; t77.kasa@gmail.com

**Keywords:** portable navigation, leg lengthening, offset, total hip arthroplasty

## Abstract

*Background and Objectives*: Total hip arthroplasty (THA) requires accurate implant placement to ensure optimal outcomes. In this study, the AR Hip navigation system, an imageless portable navigation tool using augmented reality (AR), was evaluated for measuring radiographic inclination (RI), anteversion (RA), leg lengthening (LL), and offset (OS) changes in supine and lateral decubitus THA. Notably, this is the first report to assess the accuracy of LL and OS measurements using AR technology. *Methods*: We analyzed 48 hips from primary THA patients: 17 in the supine (S) group and 31 in the lateral (L) group. RI, RA, LL, and OS were measured intraoperatively using AR Hip and postoperatively using Zed Hip 3D software (Version 18.0.0.0). The absolute errors and outlier rates (≥5° for RI/RA and ≥5 mm for LL/OS) were compared between groups. *Results*: The mean intraoperative RI values with AR Hip were 40.1 ± 0.6° (S), 40.2 ± 1.2° (L), and 40.1 ± 1.0° (total), while the postoperative RI values with Zed Hip were 39.7 ± 2.9° (S), 39.5 ± 2.5° (L), and 39.6 ± 2.6° (total). The absolute errors were 1.8 ± 1.7° (total), with no significant group differences (*p* = 0.957). For RA, the errors were 2.0 ± 1.2° (total) (*p* = 0.771). The LL errors were 2.3 ± 2.2 mm (total) (*p* = 0.271), and the OS errors were 3.5 ± 2.8 mm (total) (*p* = 0.620). The outlier rates for RI were 11.8% (S) and 3.2% (L); for RA, 0% (S) and 3.2% (L); for LL, 29.4% (S) and 6.5% (L) with a significant difference (*p* = 0.031); and for OS, 23.5% (S) and 25.8% (L). No significant differences were observed for RI, RA, or OS. *Conclusions*: AR Hip provided accurate measurements of cup orientation, LL, and OS in both supine and lateral THA. Importantly, this study is the first to report the accuracy of LL and OS measurements using AR technology, demonstrating the potential of AR Hip for improving THA precision.

## 1. Introduction

Total hip arthroplasty (THA) is one of the most effective treatments for hip disorders and is well known to significantly improve patients’ quality of life [1]. However, despite the remarkable success of THA, several challenges persist. Suboptimal implant positioning can adversely affect both short- and long-term postoperative outcomes. Improper cup alignment increases the risk of impingement, dislocation, and edge-loading, which can accelerate polyethylene wear [2,3,4]. Additionally, a failure to achieve the planned leg length extension or offset correction can result in postoperative complications such as paralysis or limping, leading to decreased patient satisfaction. In some cases, these issues may lead to legal disputes, placing additional burdens not only on the patient but also on the surgeon [5,6].

Even when the target implant positioning, angles, and leg length adjustments are well understood, achieving high reproducibility in surgery can be difficult, particularly under the constraints of intraoperative pelvic movement and limited surgical fields [7,8,9]. Even experienced surgeons can face challenges in consistently performing successful surgeries under these conditions.

In this context, computer-assisted surgery (CAS) tools are increasingly being utilized in THA to enhance surgical accuracy. In Japan, the approval and introduction of CT-based navigation in 2001 marked the beginning of CAS adoption, and various tools, including robotics, are now available. Among these, imageless portable navigation systems have rapidly gained popularity in recent years due to their low cost, ease of use, and high reproducibility.

Since 2021, our institution has adopted an imageless portable navigation system, the AR Hip navigation system (AR Hip, Zimmer Biomet, Japan), for use in THA. This system utilizes augmented reality (AR) technology, allowing for real-time visual feedback through a monitor during surgery. A key feature of the system is its use of the functional pelvic plane (FPP), which includes both anterior superior iliac spines (ASISs) as reference points. The “flip technique” enables parameters to be measured in the lateral decubitus position based on FPP registration performed in the supine position [10,11].

While several validation studies have been conducted, primarily by the developers’ institutions, there have been relatively few reports on the accuracy of cup placement with this system [12,13,14,15]. The current commercially available AR Hip includes functionality for measuring cup orientation, and the prototype also features capabilities for measuring leg lengthening (LL) and offset (OS) changes. At our institution, we have been using this additional function in a limited capacity.

In this study, we retrospectively evaluated primary THAs performed at our institution using AR Hip (Version 1.0.54). The primary objectives were to assess (1) cup orientation, (2) LL and OS, and (3) the incidence of outliers. Additionally, we aimed to evaluate the impact of surgical position (supine vs. lateral decubitus) on these outcomes.

## 2. Materials and Methods

### 2.1. Participants and Design

This study included 88 primary THAs performed at our institution between January and August 2022. Among these, 48 hips from 46 patients were analyzed for cup positioning angles, LL, and OS using AR Hip. All of the surgeries were performed by two orthopedic surgeons each with over 10 years of experience (T.Y. and T.K.).

Patients were divided into two groups based on the surgical position assessed by AR Hip. The S group consisted of 15 patients (17 hips, 5 males) who underwent THA via the anterolateral approach in the supine position. The L group consisted of 31 patients (31 hips, 3 males) who underwent THA via the posterolateral approach in the lateral decubitus position. Although the S group included two cases of bilateral simultaneous THA, the AR Hip measurements were conducted separately for each hip.

The mean age at the time of surgery was 70 years (range, 39–89 years) in the S group and 68 years (range, 49–85 years) in the L group. The demographic characteristics of the patients are summarized in Table 1.

This study was conducted in accordance with the Declaration of Helsinki and was approved by the institutional ethics committee of St. Marianna University (No. 6018; the approval date was 10 July 2023). Due to the retrospective nature of this study, the requirement for obtaining written informed consent from individual patients was waived. Instead, an opt-out approach was implemented, whereby information about the study was made publicly available through the institution’s website, allowing patients to decline participation if they so wished. No patients objected to the use of their data.

### 2.2. AR Hip Navigation System (Figure 1a)

**Figure 1:**
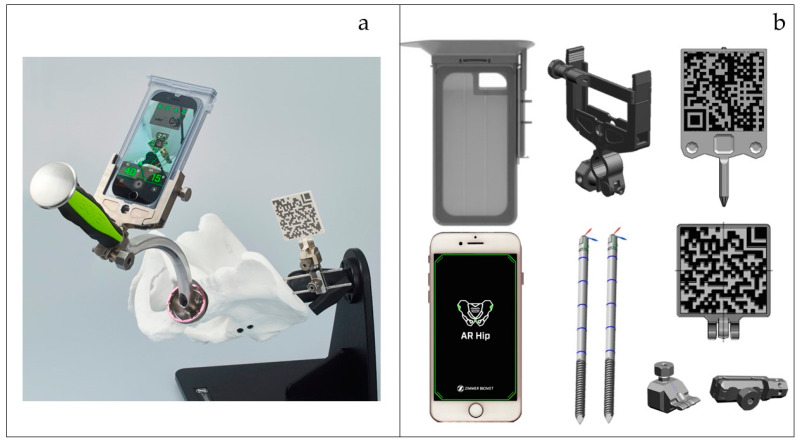
(**a**) AR Hip navigation system (Zimmer Biomet Japan). (**b**) AR Hip component machine: iPhone and its case, 3.2 mm half pin, cup impactor attachment, base holder, and various probes.

AR Hip is an imageless portable navigation system that employs augmented reality technology [12]. The iPhone camera is employed to recognize QR codes, which then project a virtual FPP grid onto the screen. During THA, the system calculates coordinates based on the sizes and angles of the markers observed by the camera, thereby enabling real-time position tracking and adjustment of the cup orientation. AR Hip is compact, consisting only of an iPhone, several QR markers, fixation pins, and holders, thus facilitating simplicity and efficiency (Figure 1b). Currently, it is widely utilized for determining cup orientation angles (abduction and anteversion) relative to the FPP; however, the prototype also includes the capability to measure LL and OS.

#### 2.2.1. AR Hip: Registration and Flip Technique

**Figure 2:**
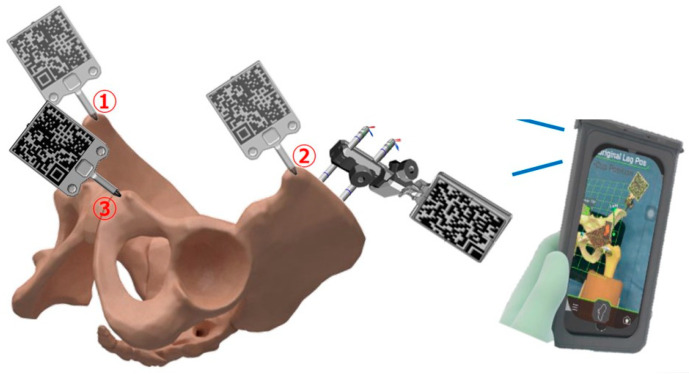
The registration process begins with ① the right ASIS, followed by ② the left ASIS and ③ the pubic symphysis. To prevent misinterpretation of the QR code, the surface facing the camera is avoided during registration.

First, two 3.2 mm half-pins are inserted into the iliac crest in the supine position. The pins are inserted posterior to the ASIS to avoid damage to the lateral femoral cutaneous nerve, and a base holder is attached to secure the QR marker. Next, the dedicated iPhone app is launched, and the pelvic marker is attached to the base holder. When the iPhone camera recognizes the pelvic QR marker, the FPP grid is projected. The camera is held at a fixed distance from the pelvic marker, and using the probe QR for registration, the right ASIS, left ASIS, and pubic symphysis are registered (Figure 2).

When recognizing the marker, it is recommended to approach it from an angle rather than directly in front to avoid errors. Additionally, caution should be taken as strong light sources, such as surgical lamps, may interfere with the camera’s functionality. After registration is complete, re-reading the pelvic QR marker will display the FPP grid, including both ASISs, aligned parallel to the ground. When the cup orientation screen is accessed, the acquired angles within the FPP are displayed.

**Figure 3:**
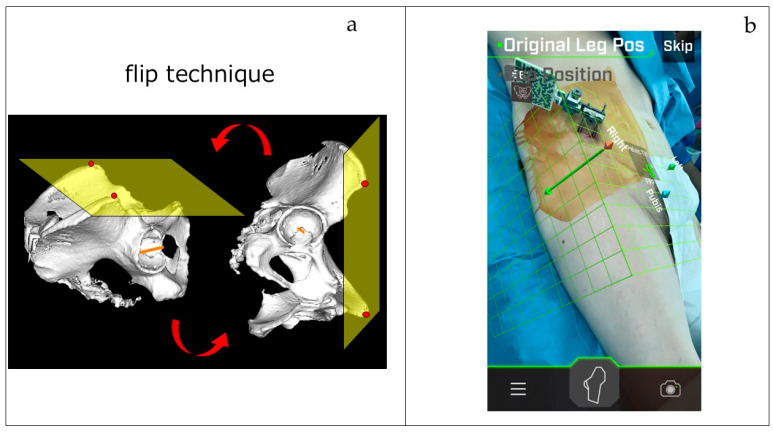
(**a**) Flip technique: the FPP grid, when measured in the supine position, is accurately reproduced as a plane perpendicular to the ground by 90 degrees in the lateral decubitus position when the flip technique is employed. (**b**) Both the supine and lateral decubitus FPP grids are displayed on the actual operating screen of the iPhone.

In the lateral decubitus position, the same procedure is followed to fix the pins and complete registration, after which the patient is carefully turned to the lateral position. The pelvic stabilizer is used to ensure that the pins remain secure. The FPP grid used in the lateral position is displayed in real time on the screen and is aligned perpendicular to the FPP grid created in the supine position. Even in the lateral position, the measured parameters, such as cup orientation angles, are calculated based on the position information registered in the supine position, utilizing the so-called “flip technique” (Figure 3a,b), which has been reported to provide cup placement accuracy comparable to that of the supine system [13,14,15].

The accuracy of the measured cup orientation can be intuitively verified by checking whether the FPP grid displayed after reading the pelvic QR marker is correctly aligned. Additionally, during cup fixation, the operator can monitor the changing angles on the iPhone screen while hammering, which ensures excellent reproducibility of the target cup orientation and provides superior operability.

#### 2.2.2. Registration and Measurement for Leg Lengthening (LL) and Offset (OS) Changes

**Figure 4:**
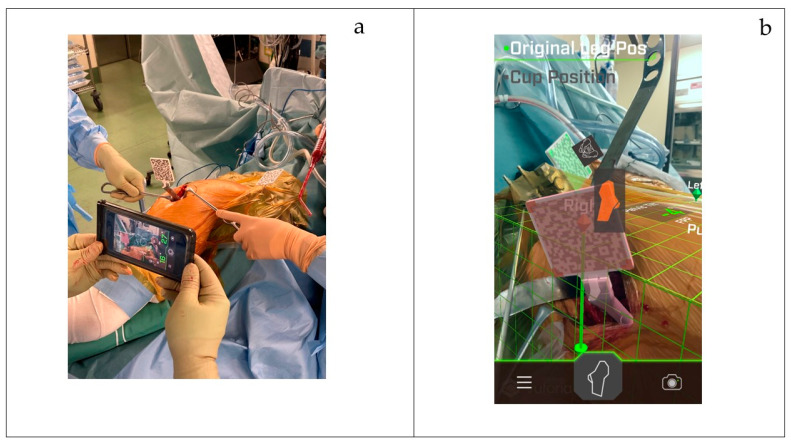
(**a**) The femoral QR marker, connected to the screw attachment, is then recognized within the pre-set FPP grid, registering the baseline position. (**b**) The femoral QR marker is registered within the FPP grid projected on the monitor. To facilitate post-implantation measurements, it is recommended to position both QR markers in such a way that they can be consistently and easily captured by the iPhone.

After completing the AR Hip registration, the surgery is initiated. Before performing the femoral neck osteotomy, a screw attachment is placed distally and anteriorly to the greater trochanter to fix the femoral QR marker in supine position surgeries. Similarly, in lateral decubitus surgeries, the attachment is placed distally to the greater trochanter. These attachments must be securely fixed in a position where they are not interfered with by skin or soft tissue. The femoral QR marker, connected to the screw attachment, is then recognized within the pre-set FPP grid, registering the baseline position (Figure 4a,b).

**Figure 5:**
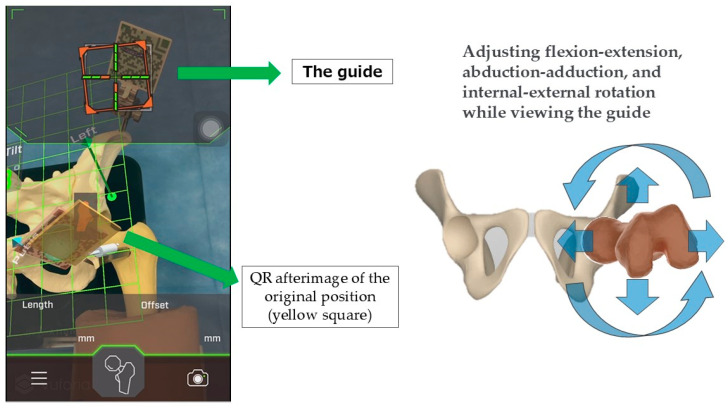
Method for measuring LL and OS: after reduction, the QR marker is reattached to the femur, and the leg is adjusted to the original position for measurement.

**Figure 6:**
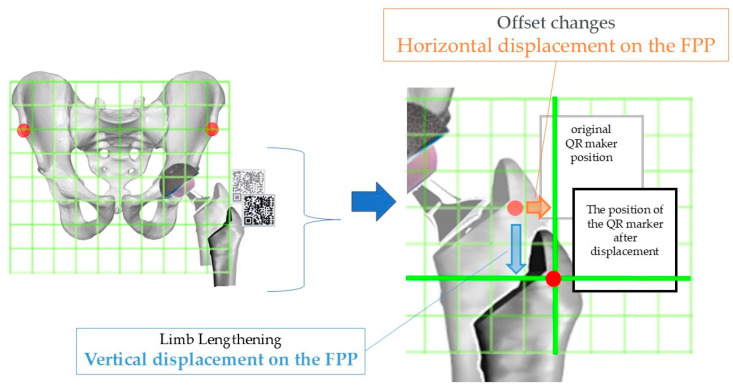
Algorithm for measuring LL and OS: When the original position and the femoral QR marker overlap, the measurement values are displayed. The amount of leg lengthening is measured as the vertical displacement on the FPP grid relative to the original position, and the offset change is measured as the horizontal displacement in the same grid.

Next, after the rasp trial or stem is inserted and the joint is reduced, the actual changes in LL and OS are measured. The femoral QR marker is reattached, and the leg is moved to replicate the original registered position. Using the guidance displayed on the iPhone, the leg is gradually adjusted through flexion, extension, abduction, adduction, and internal/external rotation until the correct position is achieved (Figure 5). Once the baseline position is replicated, the QR markers align, a confirmation sound is triggered, and the LL is displayed as a positive value in the distal vertical direction within the FPP grid. Simultaneously, the OS is displayed as a positive value along the horizontal plane, parallel to both ASISs, representing the change in offset from the pelvis (Figure 6).

### 2.3. Surgical Procedure

THA was performed using an anterolateral approach in the supine position for the S group and a posterolateral approach for the L group. In both groups, after the AR Hip registration was completed under anesthesia, the patient was positioned, and the navigation holder was sterilized along with the surgical field. The iPhone was placed in a dedicated sterile cover and used in the surgical field. In both groups, the cup was implanted first, followed by the stem. Fluoroscopy was briefly used in all cases. Fluoroscopy was performed during final acetabular reaming to confirm medialization and during the rasp trial to visually confirm alignment, LL, and OS.

The cup was positioned relative to the FPP with a target of 40 degrees of radiographic inclination (RI) and 20 degrees of radiographic anteversion (RA) based on radiographic definitions [16]. Measurements with AR Hip were taken during cup impaction, as well as during the measurement of LL and OS.

### 2.4. Postoperative Evaluation

For each case, the intraoperative values of RI, RA, LL, and OS displayed by AR Hip were recorded. Similarly, within two weeks postoperatively, CT scans taken in the supine position were analyzed using the 3D software Zed Hip (Zed Hip, Version 18.0.0.0, Lexi Co., Ltd., Tokyo, Japan) to measure RI, RA, LL, and OS. The following points were evaluated:

(1) RI and RA

The mean ± SD and range of RI and RA were calculated for group S, group L, and all 48 hips. Additionally, statistical analysis was conducted to determine if there were significant differences in absolute error between the two groups.

(2) LL and OS

The mean ± SD and range of LL and OS were calculated for group S, group L, and all 48 hips. Statistical analysis was also performed to assess whether there were significant differences in absolute error between the two groups.

(3) Calculation of Outliers

Outliers were defined as an absolute error greater than 5 degrees for RI and RA and greater than 5 mm for LL and OS. The outlier rates were calculated for both groups and for the entire cohort. Statistical analysis was conducted to determine if there were significant differences between the two groups for each parameter.

### 2.5. Statistical Analysis

Statistical analyses were performed using JMP version 16.2.0 (SAS Institute Inc., Cary, NC, USA). The Student’s *t*-test and chi-square test were used to perform statistical comparisons of demographic characteristics between the two groups. The Mann–Whitney U test was applied to compare the two groups for the four parameters—RI, RA, LL, and OS—while the chi-square test was used to assess differences in the incidence of outliers between the two groups.

## 3. Results

The average age at the time of surgery was 70 years (39–89 years) for the S group and 68 years (49–85 years) for the L group. No significant differences were observed between the two groups in terms of sex, average age, height, weight, side affected, or BMI (Table 1).

(1) RI and RA (Table 2 and Table 3)

For RI, the values measured by AR Hip were 40.1 ± 0.6° (39.0–41.0°) for group S, 40.2 ± 1.2° (38.0–45.0°) for group L, and 40.1 ± 1.0° (38.0–45.0°) for the total cohort. Postoperative measurements by Zed Hip were 39.7 ± 2.9° (32.1–45.7°) for group S, 39.5 ± 2.5° (34.5–46.9°) for group L, and 39.6 ± 2.6° (32.1–46.9°) for the total cohort. The absolute error was 2.0 ± 2.2° (0–7.9°) for group S, 1.7 ± 1.4° (0.1–6.5°) for group L, and 1.8 ± 1.7° (0–7.9°) for the total cohort, with no statistically significant difference between the two groups (*p* = 0.957).

For RA, AR Hip measured 20.8 ± 1.9° (17.0–24.0°) for group S, 21.4 ± 1.6° (18.0–25.0°) for group L, and 21.2 ± 1.7° (17.0–25.0°) for the total cohort. Postoperative measurements by Zed Hip were 20.1 ± 1.6° (17.3–23.8°) for group S, 21.0 ± 2.4° (15.4–25.0°) for group L, and 20.7 ± 2.2° (15.4–25.0°) for the total cohort. The absolute error was 2.1 ± 1.2° (0.2–4.1°) for group S, 2.0 ± 1.3° (0.1–5.4°) for group L, and 2.0 ± 1.2° (0.1–5.4°) for the total cohort, with no statistically significant difference between the two groups (*p* = 0.771).

(2) LL and OS (Table 4 and Table 5)

For LL, AR Hip measurements were 7.8 ± 4.3 mm (2.0–16.0 mm) for group S, 9.3 ± 6.2 mm (2.0–27.0 mm) for group L, and 8.8 ± 5.6 mm (2.0–27.0 mm) for the total cohort. Postoperative measurements by Zed Hip were 4.9 ± 4.8 mm (−1.4–15.5 mm) for group S, 10.3 ± 6.7 mm (2.0–30.3 mm) for group L, and 8.4 ± 6.5 mm (−1.4–30.3 mm) for the total cohort. The absolute error was 3.4 ± 2.7 mm (0–8.4 mm) for group S, 2.3 ± 1.7 mm (0.3–7.0 mm) for group L, and 2.3 ± 2.2 mm (0–8.4 mm) for the total cohort, with no statistically significant difference between the two groups (*p* = 0.271).

For OS, AR Hip measurements were −3.3 ±5.1 mm (−10.0–6.0 mm) for group S, −11.7 ± 6.9 mm (−30.0–2.0 mm) for group L, and −8.7 ± 5.6 mm (−30.0–6.0 mm) for the total cohort. The postoperative measurements by Zed Hip were −5.5 ± 5.7 mm (−14.8–5.5) mm for group S, −11.8 ± 5.7 mm (−25.0 to −3.1) for group L, and −9.6 ± 6.4 mm (−25.0–5.5) mm for the total cohort. The absolute error was 3.7 ±3.1 mm (0.1–11.4 mm) for group S, 3.3 ± 2.7 mm (0–9.4 mm) for group L, and 3.5 ± 2.8 mm (0–11.4 mm) for the total cohort, with no statistically significant difference between the two groups (*p* = 0.620).

(3) Outlier Calculation (Table 6)

For outliers, the rate of RI outliers was 11.8% for group S, 3.2% for group L, and 6.0% overall. For RA, the rates were 0% for group S, 3.2% for group L, and 2.0% overall. LL outliers were 29.4% for group S, 6.5% for group L, and 14.6% overall. OS outliers were 23.5% for group S, 25.8% for group L, and 25.0% overall. No significant differences were observed for RI, RA, or OS. However, there was a significant difference between the two groups regarding LL (*p* = 0.031).

## 4. Discussion

The objective of this study was to evaluate the accuracy of a portable navigation system that employed AR technology. It is noteworthy that there have been few reports in the literature that directly compare the accuracy of the system when used in the supine and lateral positions in THA. Furthermore, this study is notable for its assessment of the prototype’s capacity to measure LL and OS using AR Hip. To the best of our knowledge, this is the first report globally to provide empirical measurements of LL and OS using AR technology.

Computer-assisted orthopedic surgery (CAOS) has gradually become a widely accepted approach in the field of THA, offering the potential for more precise and optimal surgical outcomes. The advent of navigation technology has been associated with enhanced accuracy in implant placement and improved clinical outcomes [17,18]. Nevertheless, from a broader perspective on joint replacement surgery, there is currently insufficient evidence to definitively demonstrate the effectiveness of CAOS in terms of long-term outcomes, operative time, and cost-effectiveness for THA [18,19].

It has been reported that portable navigation systems, such as AR Hip, have lower implementation costs in comparison with CT-based navigation systems [20,21,22]. The cost of using AR Hip is similarly low, approximately USD 600 per case, with an additional government subsidy of around USD 200 in Japan, which serves to further reduce the barrier to adoption. This cost-effectiveness makes it an attractive option for institutions, and these factors support its utility as an adjunctive tool in THA. Furthermore, even in countries or regions with limited medical resources, this system could play a crucial role in improving the outcomes of THA procedures.

Prior studies have demonstrated the accuracy of cup placement using AR-based portable navigation systems, with absolute errors of approximately 2 to 3 degrees for both RI and RA [13,14,15,22]. In this study, the accuracy of cup placement using AR Hip demonstrated a mean absolute error of 1.8 ± 1.7° (0–7.9°) for RI and 2.0 ± 1.2° (0.1–5.4°) for RA across all cases, which was comparable to or better than previous reports.

These findings exceed those reported for portable navigation systems that utilize the anterior pelvic plane (APP) as the reference plane [23]. The utilization of the APP as a reference plane in navigation systems often results in reduced registration accuracy due to the presence of thick subcutaneous tissue overlying the pubic symphysis, which is included as a landmark in APP-based systems [24].

In contrast, AR Hip employs the FPP, which is derived from both the ASISs and the gravitational direction, thereby reducing registration errors caused by soft tissue. The performance of these systems in supine THA also exhibits favorable outcomes, with absolute errors for the range of inclination (RI) and the anteversion angle (RA) generally within the range of 2 to 3 degrees [25].

In the context of lateral position THA, where substantial alterations in body position are anticipated, there is a substantial opportunity for the utilization of intraoperative support tools such as CAS. However, the process of acquiring landmarks in the lateral position has proven to be a significant challenge. AR Hip addresses this issue by employing the “flip technique”, which entails applying the FPP reference obtained during supine registration to lateral position THA. The results demonstrate that the outlier rate, defined as deviations exceeding 5.1°, was 6.0% for RI and 2.0% for RA, with no statistically significant difference between the two surgical positions. These findings indicate that the surgical position does not affect the accuracy of AR Hip, demonstrating that the FPP registration performed in the supine position can be reliably applied to lateral position THA.

Su et al. reached the conclusion in a systematic review and meta-analysis of five studies that AR navigation offers superior cup placement accuracy compared with conventional methods [26]. Our results also indicate that AR Hip can be concluded to be a CAS tool capable of reproducing the cup placement in THA with a high level of precision.

A residual discrepancy in leg length following THA can result in postoperative instability, adjacent joint disorders, lumbar pain, and gait disturbances. These complications not only diminish patient satisfaction but also elevate the risk of medical litigation [27]. Similarly, offset is a pivotal element influencing dislocation resistance, gluteal muscle strength, and range of motion. It has been documented that an elevated offset in comparison with the contralateral side can result in thigh discomfort, underscoring its significance as a crucial factor [28]. The capacity to make intraoperative measurements of LL and OS and then reference them using navigation tools is therefore of great value [29].

In this study, AR Hip demonstrated high intraoperative accuracy, with absolute errors of 2.3 ± 2.2 mm (0–8.4 mm) for LL and 3.5 ± 2.8 mm (0–11.4 mm) for OS across all cases. However, the outlier rate for deviations greater than 5.1 mm was 14.6% for LL and 25.0% for OS. While there was no significant difference between the two groups in terms of absolute errors for LL and OS, a significant difference was observed between the groups in the outlier rate for LL (*p* = 0.031). One potential explanation is that in supine THA, the femoral marker holder is situated in closer proximity to the skin and soft tissue, which may result in interference and subsequent marker displacement. Furthermore, attaining full leg extension in the supine position can be challenging, making it difficult to accurately replicate the leg position during registration, which may have contributed to the larger absolute error.

While there is no consensus regarding the acceptable range of leg length discrepancy [30], discrepancies exceeding 10 mm have been found to be associated with symptoms such as limping, pelvic tilt, the need for shoe lifts, and a sense of disappointment [31,32]. Accordingly, it is imperative to restrict the margin of error to a maximum of 10 mm. In our study, no cases exhibited an absolute LL error exceeding 10 mm, indicating that AR Hip demonstrates sufficient accuracy to meet clinical expectations.

In regard to offset, it has been documented that both excessive and insufficient correction can impact clinical outcomes and polyethylene wear [28,32,33,34]. AR Hip quantifies offset as the change in a parameter parallel to both ASISs within the FPP grid; however, it does not assess anterior–posterior offset displacement. The 25% outlier rate for offset may be somewhat elevated when determining whether the offset has been adjusted in accordance with the preoperative plan. It would be prudent to utilize intraoperative fluoroscopy in conjunction with the numerical data to confirm accuracy.

Previous evaluations of the intraoperative measurement capabilities for LL and OS using imageless portable navigation systems (Table 7 [29,35,36]) have typically employed plain radiographs for postoperative measurements, with an estimated error margin of approximately 2–3 mm for both LL and OS. The LL and OS measurement function of AR Hip (prototype) has been demonstrated to exhibit a similar level of accuracy to that of these systems. The findings of this study indicate that the measurement functions for LL and OS are significantly more reliable than visual confirmation alone, suggesting that AR Hip has strong potential for practical application. However, further improvements in ease of use and reproducibility are expected.

The limitations of this study include the small number of cases and the fact that the coordinate axes used for measuring errors differ slightly between AR Hip and the postoperative CT analysis using the Zed Hip system, which may have influenced the results.

## 5. Conclusions

The results of this study indicated that the portable navigation system employing AR technology exhibited accuracy in cup placement that was comparable to or superior to that of existing imageless portable navigation systems. Furthermore, the system demonstrated consistent precision in both the supine and lateral positions. The prototype’s capacity to measure LL and OS was also found to be comparable to that of existing imageless systems, falling within clinically acceptable limits. These findings suggest that AR-based portable navigation offers a valuable tool for enhancing implant positioning accuracy in total hip arthroplasty.

## Figures and Tables

**Table 1:** Patients’ demographic characteristics.

	Group S (Supine)	Group L (Lateral)	Total	Group S vs. Group L *p*-Value
Number of hips	17	31	48	
Sex, male/female	5/10	4/27	9/37	0.215 **
	Fe: 67%	Fe: 87%		
Age (y) ± SD	70 ± 14	68 ± 10.6	68.7 ± 11.8	0.570 *
Height (m) ± SD	1.59 ± 0.07	1.54 ± 0.07	1.55 ± 0.07	0.470 *
Weight (kg) ± SD	56.4 ± 10.1	62.1 ± 10.3	60.1 ± 10.5	0.700 *
Mean BMI	23.2	26.1	25.1 ± 3.96	0.120 *
Side affected, left/right	6/11	13/18	19/29	0.084 **
Primary diagnosis				
OA	12 (71%)	29 (94%)	41	
ON	5	2	7	

BMI: body mass index; OA: osteoarthritis; ON: osteonecrosis. Results are expressed as mean ± SD. * *p* values were determined with Student’s *t*-test. ** *p* values were determined with chi-square test.

**Table 2:** Intraoperative measurements with AR Hip and postoperative CT analysis with Zed Hip for RI and absolute value errors.

RI	Group S (*n* = 17)	Group L (*n* = 31)	Total (*n* = 48)	Group S vs. Group L *p*-Value
AR Hip (°)	40.1 ± 0.6 (39.0–41.0)	40.2 ± 1.2 (38.0–45.0)	40.1 ± 1.0 (38.0–45.0)	
Zed Hip (°)	39.7 ± 2.9 (32.1–45.7)	39.5 ± 2.5 (34.5–46.9)	39.6 ± 2.6 (32.1–46.9)	
Absolute error (°)	2.0 ± 2.2 (0–7.9)	1.7 ± 1.4 (0.1–6.5)	1.8 ± 1.7 (0–7.9)	0.957 *

Results are expressed as mean ± SD. * *p* values were determined with the Mann–Whitney U test.

**Table 3:** Intraoperative measurements with AR Hip and postoperative CT analysis with Zed Hip for RA and absolute value errors.

RA	Group S (*n* = 17)	Group L (*n* = 31)	Total (*n* = 48)	Group S vs. Group L *p*-Value
AR Hip (°)	20.8 ± 1.9 (17.0–24.0)	21.4 ± 1.6 (18.0–25.0)	21.2 ± 1.7 (17.0–25.0)	
Zed Hip (°)	20.1 ± 1.6 (17.3–23.8)	21.0 ± 2.4 (15.4–25)	20.7 ± 2.2 (15.4–25.0)	
Absolute error (°)	2.1 ± 1.2 (0.2–4.1)	2.0 ± 1.3 (0.1–5.4)	2.0 ± 1.2 (0.1–5.4)	0.771 *

Results are expressed as mean ± SD. * *p* values were determined with the Mann–Whitney U test.

**Table 4:** Intraoperative measurements with AR Hip and postoperative CT analysis with Zed Hip for LL and absolute value errors.

LL	Group S (*n* = 17)	Group L (*n* = 31)	Total (*n* = 48)	Group S vs. Group L *p*-Value
AR Hip (mm)	7.8 ± 4.3 (2.0–16.0)	9.3 ± 6.2 (2.0–27.0)	8.8 ± 5.6 (2.0–27.0)	
Zed Hip (mm)	4.9 ± 4.8 (−1.4–15.5)	10.3 ± 6.7 (2.0–30.3)	8.4 ± 6.5 (−1.4–30.3)	
Absolute error (mm)	3.4 ± 2.7 (0–8.4)	2.3 ± 1.7 (0.3–7.0)	2.3 ± 2.2 (0–8.4)	0.271 *

Results are expressed as mean ± SD, range. * *p* values were determined with the Mann–Whitney U test.

**Table 5:** Intraoperative measurements with AR Hip and postoperative CT analysis with Zed Hip for OS and absolute value errors.

OS	Group S (*n* = 17)	Group L (*n* = 31)	Total (*n* = 48)	Group S vs. Group L *p*-Value
AR Hip (mm)	−3.3 ± 5.1 (−10.0–6.0)	−11.7 ± 6.9 (−30.0–2.0)	−8.7 ± 5.6 (−30.0–6.0)	
Zed Hip (mm)	−5.5 ± 5.7 (−14.8–5.5)	−11.8 ± 5.7 (−25.0–3.1)	−9.6 ± 6.4 (−25.0–5.5)	
Absolute error (mm)	3.7 ± 3.1 (0.1–11.4)	3.3 ± 2.7 (0–9.4)	3.5 ± 2.8 (0–11.4)	0.620 *

Results are expressed as mean ± SD, range. * *p* values were determined with the Mann–Whitney U test.

**Table 6:** Incidence of outliers in the four parameters RI, RA, LL, and OS in total and in both groups. Outliers were defined as ≥5.1° for RI and RA and ≥5.1 mm for LL and OS.

	Group S (*n* = 17)	Group L (*n* = 31)	Total (*n* = 48)	Chi-Square Value	Group S vs. Group L *p*-Value
RI	11.80%	3.20%	6.00%	1.37	0.242 *
RA	0%	3.20%	2.00%	0.56	0.454 *
LL	29.40%	6.50%	14.60%	4.64	0.031 *
OS	23.50%	25.80%	25.00%	0.03	0.862 *

* *p* values were determined with the chi-square test.

**Table 7:** Absolute error of LL and OS change measurements using various imageless portable navigation systems.

Author	Year	Number	Device	Study Design	Operative Position	Postoperative Evaluation	LL Absolute Error (Mean ± SD) mm	OS Absolute Error (Mean ± SD) mm
Weber M et al. [29]	2014	55	Hip6.0 prototype	RCT	Lateral	XR	1.8 ± 0.2	1.4 ± 0.2
Tanino H et al. [35]	2021	60	NewHipAlign	NP	Lateral	XR	2.3 ± 2.6	
Anjiki K et al. [36]	2022	31	HipAlign	NP	Supine	XR	3.1 ± 2.5	
This study	2024	48	AR Hip	NP	Supine (17) Lateral (31)	CT	3.4 ± 2.7 2.3 ± 1.7	3.7 ± 3.1 3.3 ± 2.7

RCT, randomized control trial; NP, nonrandomized prospective; XR, X-ray; CT, computed tomography.

## Data Availability

The data presented in this study are available on request from the corresponding author. The data are not publicly available due to privacy of patient data.

## References

[1] Learmonth I.D., Young C., Rorabeck C. (2007). The operation of the century: Total hip replacement. Lancet.

[2] Miki H., Kyo T., Kuroda Y., Nakahara I., Sugano N. (2014). Risk of edge-loading and prosthesis impingement due to posterior pelvic tilting after total hip arthroplasty. Clin. Biomech..

[3] Kennedy J.G., Rogers W.B., Soffe K.E., Sullivan R.J., Griffen D.G., Sheehan L.J. (1998). Effect of acetabular component orientation on recurrent dislocation, pelvic osteolysis, polyethylene wear, and component migration. J. Arthroplast..

[4] Lewinnek G.E., Lewis J.L., Tarr R., Compere C.L., Zimmerman J.R. (1978). Dislocations after total hip-replacement arthroplasties. J. Bone Jt. Surg. Am..

[5] Shah M., Vieira A., Mahajan A., Agrawal L., Shah D., Surme S., Velankar A. (2023). Does intra-operative fluoroscopy significantly improve component position in a primary total hip arthroplasty? Our experience in a tertiary care hospital. Indian J. Orthop..

[6] Iwakiri K., Ohta Y., Fujii T., Minoda Y., Kobayashi A., Nakamura H. (2021). Changes in patient-perceived leg length discrepancy following total hip arthroplasty. Eur. J. Orthop. Surg. Traumatol..

[7] Asayama I., Akiyoshi Y., Naito M., Ezoe M., Ishiko T. (2004). Intraoperative pelvic motion in total hip arthroplasty. J. Arthroplast..

[8] Mouri K., Madachi A., Karita T., Sudo A. (2023). Intraoperative pelvic tilt and axial rotation during total hip arthroplasty through the direct anterior approach is affected by the acetabular retractor and cup impactor. Arthroplast. Today.

[9] Schwarzkopf R., Muir J.M., Paprosky W.G., Seymour S., Cross M.B., Vigdorchik J.M. (2017). Quantifying pelvic motion during total hip arthroplasty using a new surgical navigation device. J. Arthroplast..

[10] Iwakiri K., Kobayashi A., Ohta Y., Takaoka K. (2017). Efficacy of the anatomical-pelvic-plane positioner in total hip arthroplasty in the lateral decubitus position. J. Arthroplast..

[11] Carcangiu A., D’Arrigo C., Topa D., Alonzo R., Speranza A., De Sanctis S., Ferretti A. (2011). Reliability of cup position in navigated THA in the lateral decubitus position using the ‘flip technique’. Hip Int..

[12] Ogawa H., Hasegawa S., Tsukada S., Matsubara M. (2018). A pilot study of augmented reality technology applied to the acetabular cup placement during total hip arthroplasty. J. Arthroplast..

[13] Ogawa H., Kurosaka K., Sato A., Hirasawa N., Matsubara M., Tsukada S. (2020). Does an augmented reality-based portable navigation system improve the accuracy of acetabular component orientation during THA? A randomized controlled trial. Clin. Orthop. Relat. Res..

[14] Kurosaka K., Ogawa H., Hirasawa N., Saito M., Nakayama T., Tsukada S. (2023). Does augmented reality-based portable navigation improve the accuracy of cup placement in THA compared with accelerometer-based portable navigation? A randomized controlled trial. Clin. Orthop. Relat. Res..

[15] Tsukada S., Ogawa H., Hirasawa N., Nishino M., Aoyama H., Kurosaka K. (2022). Augmented reality- vs accelerometer-based portable navigation system to improve the accuracy of acetabular cup placement during total hip arthroplasty in the lateral decubitus position. J. Arthroplast..

[16] Murray D.W. (1993). The definition and measurement of acetabular orientation. J. Bone Jt. Surg. Br..

[17] Kelley T.C., Swank M.L. (2009). Role of navigation in total hip arthroplasty. J. Bone Jt. Surg. Am..

[18] Gandhi R., Marchie A., Farrokhyar F., Mahomed N. (2009). Computer navigation in total hip replacement: A meta-analysis. Int. Orthop..

[19] Sarrel K., Hameed D., Dubin J., Mont M.A., Jacofsky D.J., Coppolecchia A.B. (2024). Understanding economic analysis and cost-effectiveness of CT scan-guided, 3-dimensional, robotic-arm assisted lower extremity arthroplasty: A systematic review. J. Comp. Eff. Res..

[20] Tanino H., Nishida Y., Mitsutake R., Ito H. (2020). Portable accelerometer-based navigation system for cup placement of total hip arthroplasty: A prospective, randomized, controlled study. J. Arthroplast..

[21] Hayashi S., Hashimoto S., Takayama K., Matsumoto T., Kamenaga T., Fujishiro T., Hiranaka T., Niikura T., Kuroda R. (2020). Evaluation of the accuracy of acetabular cup orientation using the accelerometer-based portable navigation system. J. Orthop. Sci..

[22] Hasegawa M., Naito Y., Tone S., Sudo A. (2024). Comparison between accuracy of augmented reality computed tomography-based and portable augmented reality-based navigation systems for cup insertion in total hip arthroplasty. Sci. Rep..

[23] Ybinger T., Kumpan W. (2007). Enhanced acetabular component positioning through computer-assisted navigation. Int. Orthop..

[24] Sendtner E., Schuster T., Wörner M., Kalteis T., Grifka J., Renkawitz T. (2011). Accuracy of acetabular cup placement in computer-assisted, minimally-invasive THR in a lateral decubitus position. Int. Orthop..

[25] Tetsunaga T., Yamada K., Tetsunaga T., Sanki T., Kawamura Y., Ozaki T. (2020). An accelerometer-based navigation system provides acetabular cup orientation accuracy comparable to that of computed tomography-based navigation during total hip arthroplasty in the supine position. J. Orthop. Surg. Res..

[26] Su S., Wang R., Chen Z., Zhou F., Zhang Y. (2023). Augmented reality-assisted versus conventional total hip arthroplasty: A systematic review and meta-analysis. J. Orthop. Surg. Res..

[27] Patterson D.C., Grelsamer R.P., Bronson M.J., Moucha C.S. (2017). Lawsuits after primary and revision total hip arthroplasties: A malpractice claims analysis. J. Arthroplast..

[28] Liebs T.R., Nasser L., Herzberg W., Rüther W., Hassenpflug J. (2014). The influence of femoral offset on health-related quality of life after total hip replacement. Bone Jt. J..

[29] Weber M., Woerner M., Springorum R., Sendtner E., Hapfelmeier A., Grifka J., Renkawitz T. (2014). Fluoroscopy and imageless navigation enable an equivalent reconstruction of leg length and global and femoral offset in THA. Clin. Orthop. Relat. Res..

[30] White T.O., Dougall T.W. (2002). Arthroplasty of the hip. Leg length is not important. J. Bone Jt. Surg. Br..

[31] Ranawat C.S., Rao R.R., Rodriguez J.A., Bhende H.S. (2001). Correction of leg -length inequality during total hip arthroplasty. J. Arthroplast..

[32] Manzotti A., Cerveri P., De Momi E., Pullen C., Confalonieri N. (2011). Does computer-assisted surgery benefit leg length restoration in total hip replacement? Navigation versus conventional freehand. Int. Orthop..

[33] McGrory B.J., Morrey B.F., Cahalan T.D., An K.N., Cabanela M.E. (1995). Effect of femoral offset on range of motion and abductor muscle strength after total hip arthroplasty. J. Bone Jt. Surg. Br..

[34] Sakalkale D.P., Sharkey P.F., Eng K., Hozack W.J., Rothman R.H. (2001). Effect of femoral component offset on polyethylene wear in total hip arthroplasty. Clin. Orthop..

[35] Tanino H., Nishida Y., Mitsutake R., Ito H. (2021). Accuracy of a portable accelerometer-based navigation system for cup placement and intraoperative leg length measurement in total hip arthroplasty: A cross-sectional study. BMC Musculoskelet. Disord..

[36] Anjiki K., Kamenaga T., Hayashi S., Hashimoto S., Kuroda Y., Nakano N., Fujishiro T., Hiranaka T., Niikura T., Kuroda R. (2022). Effectiveness of an accelerometer-based portable navigation for intraoperative adjustment of leg length discrepancy in total hip arthroplasty in the supine position. J. Orthop. Sci..

